# Lung abscess predicts the surgical outcome in patients with pleural empyema

**DOI:** 10.1186/1749-8090-5-88

**Published:** 2010-10-20

**Authors:** Hung-Che Huang, Heng-Chung Chen, Hsin-Yuan Fang, Yi-Chieh Lin, Chin-Yen Wu, Ching-Yuan Cheng

**Affiliations:** 1 Department of Surgery, Changhua Christian Hospital,135 Nanshiao Street, Changhua, 500, Taiwan; 2 Department of Surgery, China Medical University Hospital, China Medical University, 2 Yude Road, Taichung, 404, Taiwan

## Abstract

**Objectives:**

Most cases of pleural empyema are caused by pulmonary infections, which are usually combined with pneumonia or lung abscess. The mortality of patients with pleural empyema remains high (up to 20%). It also contributes to higher hospital costs and longer hospital stays. We studied pleural empyema with combined lung abscess to determine if abscess was associated with mortality.

**Methods:**

From January 2004 to December 2006, we retrospectively reviewed 259 patients diagnosed with pleural empyema who received thoracscopic decortications of the pleura in a single medical center. We evaluated their clinical data and analyzed their chest computed tomography scans. Outcomes of pleural empyema were compared between groups with and without lung abscess.

**Results:**

Twenty-two pleural empyema patients had lung abscesses. Clinical data showed significantly higher incidences in the lung abscess group of pre-operative leukocytosis, need for an intensive care unit stay and mortality.

**Conclusion:**

Patients with pleural empyema and lung abscess have higher intensive care unit admission rate, higher mortality during 30 days and overall mortality than patients with pleural empyema. The odds ratio of lung abscess is 4.685. Physician shall pay more attention on high risk patient of lung abscess for early detection and management.

## Background

Pleural empyema is one of the serious complications of pneumonia, and increases the morbidity and mortality due to pneumonia [1-3]. About 5% of patients with pneumonia suffer from pleural empyema [4,5]. About 65,000 patients in the United State and the United Kingdom suffer annually from pleural empyema or a complicated parapneumonic effusion. The mortality of patients with pleural empyema is up to 20% and contributes to higher hospital costs. Inflammatory mechanisms and alterations in the balance of pleural fibrinolysis have been implicated in the pathophysiology of infectious pleural effusion. Pleural empyema is associated with fibrin deposition over pleural surfaces due to inhibition of the fibrinolysis system [6,7]. Parapneumonic effusions progress through exudative and fibrinopurulent stages and terminate in empyema in the organized stage. The clinical courses of patients with parapneumonic effusions or pleural empyema are varied. Lung abscess is defined as a circumscribed collection of pus in the lung, which leads to formation of a cavity. It develops when a localized area of parenchymal infection becomes necrotic and then cavitates. It most commonly occurs secondary to aspiration in patients with poor dentition or as a complication of necrotizing pneumonia. Lung abscess has previously been thought to be a rare condition of empyema and parapneumonic effusions. About 90% of patients with lung abscesses been cured by antibiotics therapies simply [8]. Surgical resection of lung abscess is rare when medical treatments fail.

Pleural empyema and lung abscess are both a part of low respiratory tract infection. According to the clinical observation, pleural empyema and lung abscesses may happen on the same patient. However, the strategies of these two diseases are so different. It is interesting whether the surgical results are the same of empyema patients with and without lung abscesses. We compared the clinical presentations and surgical results of patients with pleural empyema with and without lung abscesses.

## Methods

### Patients

**Figure 1:**
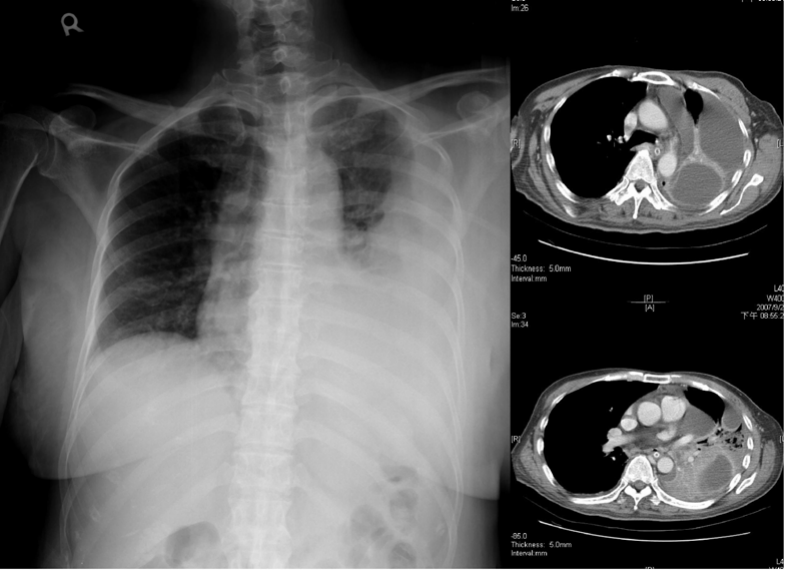
**The chest radiograph and computed tomography scan showed pleural empyema without lung abscess**.

**Figure 2:**
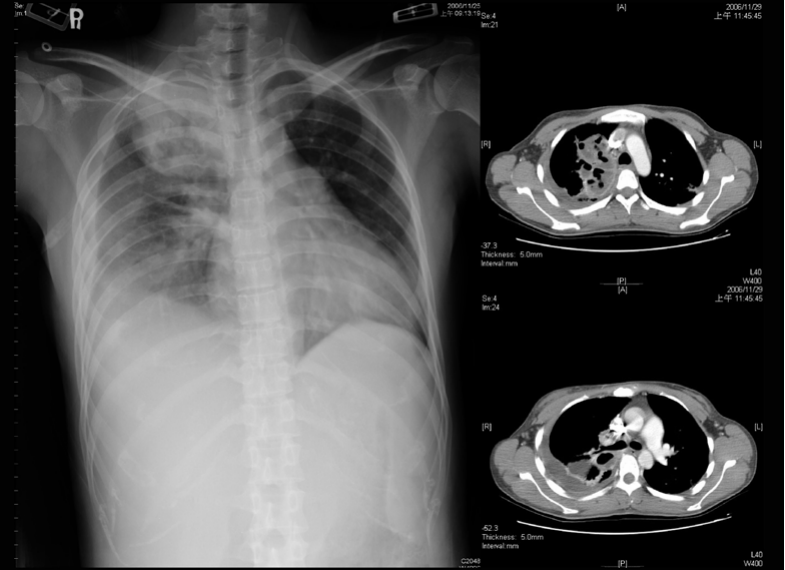
**The chest radiograph and computed tomography showed pleural empyema with lung abscess**.

This was a retrospective cohort study conducted in evaluation the impact of lung abscess on the surgical results of patients with pleural empyema. From January 2004 to December 2006, 259 patients were diagnosed with pleural empyema and received thoracoscopic decortication of pleural in Changhua Christian Hospital in central Taiwan. The diagnosis for all the patients was based initially on a chest X-ray followed by a computed tomography (CT) scan or ultrasound. All of the operations were performed by one of four qualified thoracic surgeons in our hospital. Pleural empyema was classified according to the American Thoracic Society staging; stage I is exudative pleuresia, stage II is fibrinopurulent and stage III is organized. Thoracentesis was performed on these patients for a sample of pleural fluid to determine pH, lactate dehydrogenase, glucose, protein levels, and blood cell count. After the diagnosis was established, all the patients were treated with an appropriate antibiotic therapy. The patients, who were classified into phase II or phase III pleural empyema received video-assist thoracoscopic surgery (VATS) for decortications of pleura. The operation was converted to open thoracotomy if it was failed by VATS. The patients with pleural empyema and lung abscess received VATS decortications only. We recorded the following clinical data: age, gender, and clinical findings; chronology of initial signs and diagnoses; bacteriological and biochemical studies of pleural fluids; and radiological and pre-operative findings. The vital signs were recorded just before operation in the operation room. Lung abscess was defined as a circumscribed collection of pus in the lung that led to cavity formation, which was noted on chest radiograph, CT scan or intraoperative findings by the surgeon. In our study, there was no any patient in lung abscess group received additional chest tube insertion or abscess aspiration before or after operation. Figure 1 shows an example of chest radiograph and CT scan for loculated pleural fluid collection and Figure 2 is an example of chest radiograph and CT scan of pleural empyema accompanied by abscess. Leukocytosis was defined as a white blood cell count > 10,000/μL (reference value in Changhua Christian Hospital). The outcome measures were post-operative complications and the length of hospitalization.

### Surgical procedures

All patients were transferred to the operating room and underwent general anesthesia with double-lumen endotracheal tube or single lumen endotracheal tube intubation. A patient was placed in the true lateral decubitus position on the side opposite to the empyema. Two ports were used (telescope and one instrument) after selective one lung ventilation or apnea. The wound was enlarged when the thoracoscopic procedure was difficult to perform. After a systematic sampling of fluid, abundant irrigation and aspiration were performed. Extensive debridement and ablation of all septa allowed the entire pleural cavity to be unified. Removal of the visceral and parietal pleural peel was by VATS as complete as possible, with attention paid to the visceral pleura in order to avoid air leakage. The lung re-expansion after decortication was confirmed during operation by two lung ventilation. If failure, mini-thoracotomy was performed for adequate decortication until full expansion of the lung was confirmed. Two chest tubes (28 or 32 Fr) were positioned to the anterior and posterior. After the operation, the chest tube was connected to one-bottle system that was set to a negative pressure (15 cm H_2_O using an Emerson postoperative suction pump) regularly.

### Statistical methods

Data are presented as median medians ± standard error for continuous variables and number (percentage) for categorical variables. Continuous and categorical variables were statistically compared by Mann-Whitney U test and Fisher's exact test. Survival curves were generated using the Kaplan-Meier method and differences were determined using the log-rank test. A two-tailed P-value of ≤ 0.05 was considered significant.

## Results

There were 259 patients who had pleural empyema from January 2004 to December 2006 who underwent surgical interventions during the investigation period. There were 202 (78%) men and 57 (22%) women. Nineteen patients died during the same admission. The surgical mortality rate was 7.3% (19 of 259). All early and late deaths were attributed to progressive uncontrolled sepsis.

The causes of pleural empyema included low respiratory infection (n = 239, 92%), lung cancer (n = 9, 3.5%), induced by deep neck infection (n = 1, 0.39%), post-traumatic empyema (n = 6, 2.3%) and post-operative complication (n = 4, 1.5%). Two patients were converted to mini-thoracotomy (2 of 259, 0.77%). In abscess group, there were nineteen phase II patients and three phase III patients. In non-abscess group, there were six phase I patients, two hundred and seven phase II patients and three phase III patients. There was no significant different between the two groups (P = 0.052).

Bacteria culture were performed for the 259 patients during their operations and microorganism growth was detected in 86 sets (86 of 259, 33%). There were 161 patients who had bacterial blood cultures and 25 positive results (16%); 173 patients had bacterial cultures of pleural effusion before surgery and only 42 positive results (24%).

The mean hospital stay was 24.8 ± 31.7 days and the mean post-operative hospital stay was 17.5 ± 27.8 days in all patients. The mean period of pre-operative antibiotic therapy in the mortality group was 13.0 ± 11.5 days. The mean period of pre-operative antibiotic therapy in the surviving group was 7.6 ± 9.8 days. There was a significant different between the two groups (P = 0.037). There were no significant differences in clinical presentations, such as heart rate, body temperature, mean arterial pressure, respiratory rates or co-morbidities (Table 1).

**Table 1:** Characteristics of pleural empyema patients with and without lung abscess

	Lung abscess (N = 22)	Non- lung abscess (N = 237)	P-value
Age (year)	51.9 ± 25.0	57.8 ± 18.4	P = 0.298

Gender			
Male	18	184	P = 0.792
Female	4	53	

Diabetes mellitus	5	71	P = 0.476

Cerebrovascular accident	4	26	P = 0.299

Hypertension	5	76	P = 0.366

Tuberculosis	1	25	P = 0.371

Chronic obstructive pulmonary disease	1	11	P = 1.000

Peptic ulcer	2	17	P = 0.669

Asthma	1	3	P = 0.300

Lung cancer	0	9	P = 1.000

Other malignancy	2	16	P = 0.656

Leukocytosis (WBC > 10000/mm^3^)	21	150	P = 0.002*

Heart rate (/min)	96.3 ± 18.8	94.5 ± 16.5	P = 0.418

Mean arterial pressure (mmHg)	93.1 ± 10.8	96.9 ± 14.0	P = 0.211

Body temperature (°C)	37.0 ± 0.8	37.1 ± 2.1	P = 0.947

Respiratory rate (/min)	23.0 ± 6.5	21.6 ± 5.2	P = 0.233

1. Values are medians ± standard error for continuous variables or # cases for categorical variables.

2. P-values from Mann-Whitney U test (continuous variables) or Fisher exact test (categorical variables).

**Figure 3:**
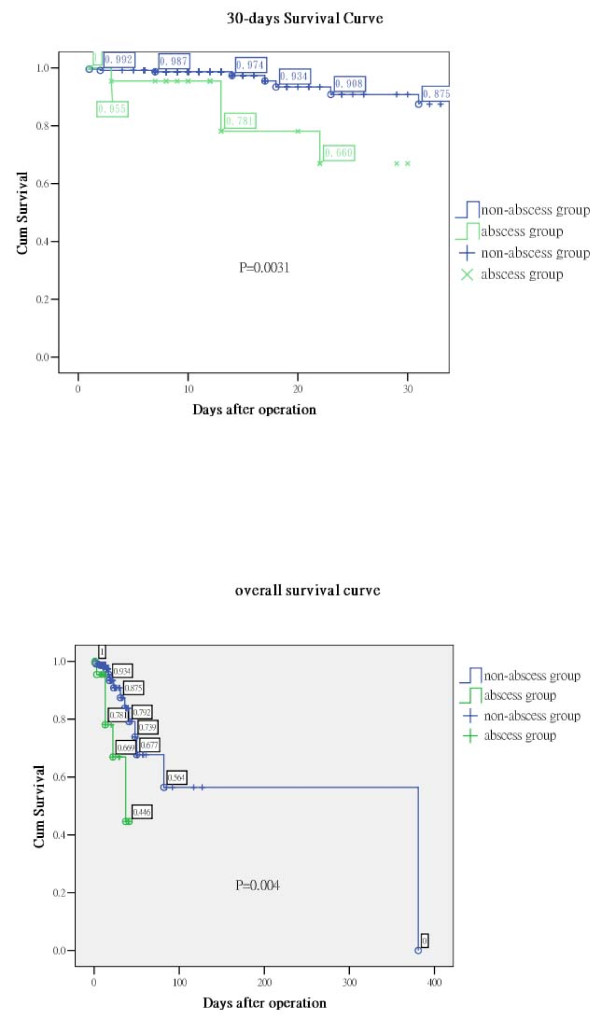
**Survival curves after surgery**. The survival curves used the Kaplan-Meier method and differences were calculated using the log-rank test. Upper panel: The 30 days survival shows a significant difference (P = 0.003). Lower panel: The overall post-operative survival between the 2 groups also shows a significant difference (P = 0.004).

There were 22 patients with lung abscesses based on image studies or by the findings during surgery. Pre-operative leukocytosis (P = 0.002), need for intensive care unit stays (P = 0.032), 30 days mortality (P = 0.003; Figure 3, upper panel) and overall mortality (P = 0.004; Figure 3, lower panel) were significantly different between the abscess group and the no abscess group (Table 2). Patients with lung abscess formation might require additional surgical procedures for residual empyema (P = 0.081).

**Table 2:** Outcomes of pleural empyema patients with and without lung abscess

	Lung abscess (N = 22)	Non-lung abscess (N = 237)	P-value
Intensive care unit admission	14(64%)	95(40%)	P = 0.032*

Length of hospital stay (days)	24.9 ± 32.8	24.8 ± 16.6	P = 0.992

Post-operation length of hospital stay (days)	17.3 ± 28.9	17.5 ± 11.0	P = 0.978

Mortality	5(23%)	14(6%)	P = 0.004*

1. Values are medians ± standard error for continuous variables or # cases (%) for categorical variables.

2. P-values from Mann-Whitney U test (continuous variables) or Fisher exact test (categorical variables).

14 patients were cared in intensive care unit before operation due to respiratory failure or unstable vital signs. In abscess group, there was only one patient cared in intensive care unit. In non-abscess group, there were 13 patients cared in intensive care unit. Excluding patients cared in intensive care unit (ICU) before operation, the patient cared in ICU after operation is 13 in abscess group and 81 in non-abscess group. The rate of admission to ICU after operation had significant different between the groups (P = 0.023).

The data of alcohol use of 194 patients were available. It was collected from patient himself, nurse record and medical chart. There were 24 patients use alcohol sometimes and 3 patients had abscess formations. 14 patients used alcohol everyday but no one had abscess formation. By the available data, the patient number of alcohol use or alcohol abuse had no significant difference between the abscess and non-abscess group (P = 0.625). There was also no significant difference between the mortality and survive group (P = 0.557).

## Discussions

About 20% of cases of paraneumonic effusion progress to pleural empyema despite the effective antibiotics and drainage of pleural effusion [3]. Early diagnosis and prompt drainage of pleural space infections are crucial, as delay increases morbidity. Pleural empyema can occur as a complication of pneumonia, tuberculosis or surgical procedures. In our study, the majority of our cases resulted from respiratory tract infection, as the same as other reports. An appropriate treatment for pleural empyema will include sepsis control, restoration of pulmonary function and prevent lung entrapment after the fibrous peel [9,10].

A lung abscess is a thick-walled cavity that contains purulent material and can occur at any age [11]. About ninety percent of patients with lung abscesses were cured by simply antibiotics therapy [8,12]. It is rarely necessary to resect the lung abscesses. The role of surgery for lung abscess is to manage the complications, including pleural empyema and bronchopleural fistula. Some patients had pleural empyema and lung abscess at the same time. In this study, the patient characteristics showed no significant differences between the two groups such as co-morbidity and clinical presentation, but leukocytosis. There were more patients with leukocytosis in lung abscess group. There were 21 (96%) patients with leukocytosis in the abscess group. Only 150 (63%) patients had leukocytosis of the patients in the no abscess group. However, leukocytosis may be related to inflammation or infection, but the number of white cell counts does not reflect the severity of inflammation or infection. Although the difference between the two group has statistical significant (P = 0.002), it is rough to conclude the diagnosis and severity according to the white cell count.

The leading cause of pleural empyema in our study was low respiratory tract infection and the incidence was 93%, and 22 of these patients (8.5%) had abscesses. Previous studies also identified bronchopulmonary infection as an important cause of empyema [13]. Lung malignancy, post-trauma, post-operative complications and deep neck infection were the other causes of pleural empyema in our study. Our study showed less post-traumatic pleural empyema rate than previous study. The low incidence may be due to early chest tube drainage when traumatic patients had related pleural effusion in our department [14].

Lung abscess have been associated with alcohol abuse. However, in our study, the patient number of alcohol use or alcohol abuse had no significant difference between the abscess and non-abscess group (P = 0.625). There was also no significant difference between the mortality and survive group (P = 0.557). However, the data was limited by the patient number of abscess group, the accuracy of medical record and nurse record, as well as the different definition of alcohol use and alcohol abuse.

The mean periods of pre-operative antibiotic therapy in the mortality group was longer than in the surviving group, respectively (P = 0.037). According to these results, early surgery after diagnosis appears to decrease the mortality. Some studies showed that early decortication of the pleura by VATS was a safe, curative treatment of pleural empyema with low morbidity [15,16]. However, some patients in our study were admitted for other diseases or were given antibiotics for other infection sources before pleural empyema was diagnosed. Longer antibiotics period may be due to poor infection control or nosocomial infection. The delay or increase duration of preoperative antibiotics may result from delaying diagnosis of empyema or lung abscess. There are many patients with low respiratory infection complicated with pleural empyema. The diagnosis shall be kept in mind. According to Coote et al and Petrakis et al, once pleural empyema is diagnosed, early and adequate drainage as well as early operation, especially the less invasive operation, VATS, is helpful to patient [17,18].

Bacterial cultures of the pleural empyema were performed in all patients. However, there were only 33% positive results from all these cultures. There were 161 patients who had bacterial blood cultures and only 25 (16%) positive results; 173 patients had bacterial cultures of pleural effusions before surgery and only 42 (24%) positive results. There was no significant difference for mortality based on the results of bacterial cultures. Echo guidance aspiration for pleural effusion is helpful to distinguish the quality of pleural effusion which is a guide of management. As Nyambat et al suggested in 2008, due to the low culture rate, culture may not be a sufficiently sensitive diagnostic method to determine the etiology in the majority of cases. The cost-effectiveness of pre-operative pleural effusion culture or blood culture shall be discussed after further study.

The abscess group also showed a higher frequency to enter the ICU after surgery (P = 0.032). After excluding the patients in ICU before operation (one in abscess group and thirteen in no abscess group), the frequency to enter the ICU after surgery still has significant difference (P = 0.023). The indications for admission to an ICU were unstable vital signs, unstable respiratory patterns and previous ICU stays. The result revealed that the patients with pleural empyema and lung abscess were more critical.

However, there was no statistical difference in the length of ICU stays, lengths of admission or length of post-operative stays. This may have been due to the large capacity of the respiratory care center or respiratory care ward. All the patients could be transferred to these units after sepsis or bronchopulmonary infections were controlled, and then transferred to a nursing home if conditions became stable and necessary. It also may be due to the failure to calculate the length of stay in other hospital before transferring to our hospital. In our study, the length of stay for patients in pleural empyema was 24.8 days and the length of stay after surgery was 17.5 days. The length of stay was longer than previous data. This may resulted from co-morbidity, delayed diagnosis of pleural empyema or a delay in surgical intervention. Some studies showed that early surgical intervention was the most optimal and cost-effective initial modality for the treatment of empyema [19].

Five (23%) patients died after their operations in the abscess group within 30 days of surgery or during the same admission. The mortality rate of the no abscess group was only 5.9%, consistent with overall mortalities observed in previous series studies [13,19,20]. Patients with abscess had a higher mortality rate than patients without lung abscess (P = 0.004). The Odds ratio for lung abscess was 4.69 (95% confidence interval = 1.057-14.56). Furthermore, patients in the abscess group also had a trend to receive second decortications of the pleura (P = 0.081). The multiple logistic regressions revealed lung abscess was not an independent predictor of death. Why the lung abscess group required further procedures? According to our data, it may be due to worse condition of the lung abscess group. The operator may stopped the operation before the completely removal of the peel due to unstable vital signs during operation. Bronchopleural fistula may also play a role in such a situation; however we had only a little experience with bronchopleural fistula. The overall mortality was higher in the abscess group, too.

## Conclusion

Patients with pleural empyema and lung abscess have higher ICU admission rate, higher mortality during 30 days and overall mortality than patients with pleural empyema. The Odds ratio of lung abscess is 4.685. Physician shall pay more attention on high risk patient of lung abscess for early detection and management.

## Competing interests

The authors declare that they have no competing interests.

## Authors' contributions

HCH carried out the manuscript. HYF designed the study and coordinated all authors. YCL and CYW collected references; HCC took the pictures of the report. CYC made conclusion. All authors read and approved the final manuscript.

## References

[B1] Schopf LF Fraga JC Amantea SL Sanches P Muller A Borowski S Kulczynski J Costa E Induction of pleural empyema in rats by thoracentesis with intrapleural pressure monitoring Pediatric surgery international 2004 20 515 519 10.1007/s00383-004-1227-2 15205903

[B2] Jess P Brynitz S Friis Moller A Mortality in thoracic empyema Scand J Thorac Cardiovasc Surg 1984 18 85 87 6719079 10.3109/14017438409099390

[B3] Sahn SA Management of complicated parapneumonic effusions Am Rev Respir Dis 1993 148 813 817 8368654 10.1164/ajrccm/148.3.813

[B4] Bouros D Plataki M Antoniou KM Parapneumonic effusion and empyema: best therapeutic approach Monaldi Arch Chest Dis 2001 56 144 148 11499304

[B5] Kunz CR Jadus MR Kukes GD Kramer F Nguyen VN Sasse SA Intrapleural injection of transforming growth factor-beta antibody inhibits pleural fibrosis in empyema Chest 2004 126 1636 1644 10.1378/chest.126.5.1636 15539738

[B6] Idell S Girard W Koenig KB McLarty J Fair DS Abnormalities of pathways of fibrin turnover in the human pleural space Am Rev Respir Dis 1991 144 187 194 2064128 10.1164/ajrccm/144.1.187

[B7] Agrenius V Chmielewska J Widstrom O Blomback M Pleural fibrinolytic activity is decreased in inflammation as demonstrated in quinacrine pleurodesis treatment of malignant pleural effusion Am Rev Respir Dis 1989 140 1381 1385 2817601 10.1164/ajrccm/140.5.1381

[B8] Erasmus JJ McAdams HP Rossi S Kelley MJ Percutaneous management of intrapulmonary air and fluid collections Radiologic clinics of North America 2000 38 385 393 10.1016/S0033-8389(05)70169-X 10765396

[B9] Jaffe A Balfour-Lynn IM Management of empyema in children Pediatric pulmonology 2005 40 148 156 10.1002/ppul.20251 15965900

[B10] Russell-Taylor M Bacterial pneumonias: management and complication Paediatric respiratory reviews 2000 1 14 20 10.1053/prrv.2000.0014 16263437

[B11] Patradoon-Ho P Fitzgerald DA Lung abscess in children Paediatric respiratory reviews 2007 8 77 84 10.1016/j.prrv.2006.10.002 17419981

[B12] Pena Grinan N Munoz Lucena F Vargas Romero J Alfageme Michavila I Umbria Dominguez S Florez Alia C Yield of percutaneous needle lung aspiration in lung abscess Chest 1990 97 69 74 10.1378/chest.97.1.69 2295263

[B13] Hsieh MJ Liu YH Chao YK Lu MS Liu HP Wu YC Lu HI Chu Y Risk factors in surgical management of thoracic empyema in elderly patients ANZ journal of surgery 2008 78 445 448 10.1111/j.1445-2197.2008.04532.x 18522563

[B14] Scherer LA Battistella FD Owings JT Aguilar MM Video-assisted thoracic surgery in the treatment of posttraumatic empyema Arch Surg 1998 133 637 641 discussion 641-632 10.1001/archsurg.133.6.637 9637463

[B15] Kosloske AM Cushing AH Shuck JM Early decortication for anaerobic empyema in children Journal of pediatric surgery 1980 15 422 426 10.1016/S0022-3468(80)80747-0 7411351

[B16] Gun F Salman T Abbasoglu L Salman N Celik A Early decortication in childhood empyema thoracis Acta chirurgica Belgica 2007 107 225 227 17515278

[B17] Coote N Surgical versus non-surgical management of pleural empyema Cochrane database of systematic reviews (Online) 2002 CD001956 12076430 10.1002/14651858.CD001956

[B18] Petrakis IE Kogerakis NE Drositis IE Lasithiotakis KG Bouros D Chalkiadakis GE Video-assisted thoracoscopic surgery for thoracic empyema: primarily, or after fibrinolytic therapy failure? American journal of surgery 2004 187 471 474 10.1016/j.amjsurg.2003.12.048 15041493

[B19] Schiza S Siafakas NM Clinical presentation and management of empyema, lung abscess and pleural effusion Current opinion in pulmonary medicine 2006 12 205 211 16582676 10.1097/01.mcp.0000219270.73180.8b

[B20] Molnar TF Current surgical treatment of thoracic empyema in adults Eur J Cardiothorac Surg 2007 32 422 430 10.1016/j.ejcts.2007.05.028 17646107

